# Sociodemographic, Clinical, Lifestyle, and Psychological Correlates of Peripheral Neuropathy among 2- to 12-Year Colorectal Cancer Survivors

**DOI:** 10.1159/000524037

**Published:** 2022-03-18

**Authors:** Dóra Révész, Cynthia S. Bonhof, Martijn J.L. Bours, Matty P. Weijenberg, Gerard Vreugdenhil, Lonneke V. van de Poll-Franse, Floortje Mols

**Affiliations:** ^a^Center of Research on Psychological Disorders and Somatic Diseases (CoRPS), Department of Medical and Clinical Psychology, Tilburg University, Tilburg, The Netherlands; ^b^Department of Research, Netherlands Comprehensive Cancer Organisation (IKNL), Utrecht, The Netherlands; ^c^Department of Epidemiology, GROW School for Oncology and Developmental Biology, Maastricht University, Maastricht, The Netherlands; ^d^Department of Internal Medicine, Maxima Medical Centre, Veldhoven, The Netherlands; ^e^Division of Psychosocial Research and Epidemiology, The Netherlands Cancer Institute, Amsterdam, The Netherlands

**Keywords:** Peripheral neuropathy, Colorectal cancer, Cancer survivors, Psychological stress, Quality of life

## Abstract

**Background:**

Peripheral neuropathy (PN) is a debilitating complication among colorectal cancer (CRC) survivors that can become chronic. No large-scale study has yet analyzed correlates in multivariable models. We did multivariable analyses to find correlates of PN.

**Methods:**

In 1,516 all-stage Dutch CRC survivors, cross-sectional data were collected on sensory, motor, autonomic, and total PN, sociodemographic (age, sex, education, employment, partner), clinical (time since diagnosis, tumor location, stage, chemotherapy, radiotherapy, comorbidities), lifestyle (alcohol, smoking, physical activity, body mass index), psychological factors (anxiety, depression, personality), and health-related quality of life (HRQoL). After multiple imputations, correlates were analyzed with linear regressions and eliminated with backward selection.

**Results:**

CRC survivors (69 years; 42% female) were on average 5 years post-diagnosis, and 28%–65% reported PN. PN was associated with older age, being male (sensory) or female (motor), shorter time since diagnosis, chemotherapy, comorbidities, anxiety, depression, and worse scores on HRQoL domains, and pain, nausea, vomiting, insomnia, constipation, and financial problems.

**Conclusions:**

In multivariable analyses, PN is affected by receiving chemotherapy, aging, sex, comorbidities, stress-related factors, and HRQoL in CRC survivors. Future PN-related studies can include these factors, and they can be examined in longitudinal studies to gain more knowledge about chronicity and severity of PN.

## Introduction

The number of colorectal cancer (CRC) survivors is increasing [1]. As a result, more CRC survivors are concerned not only about how *long* but also how *well* they survive. Peripheral neuropathy (PN) is a painful peripheral nerve injury that is common and persisting among CRC survivors [2]. Symptoms are either sensory (e.g., tingling, numbness, and aching or burning pain in the extremities), motor (e.g., weakness and cramps), or autonomic (e.g., dizziness after standing up and blurry vision) [3]. While PN symptoms reverse or improve in the majority of patients after cancer treatment, a proportion of patients experiences chronic PN [2, 3]. Due to the high prevalence of CRC combined with the lack of a well-accepted treatment or preventive strategy against PN [4], it remains a major cancer survivorship issue.

Generally, among CRC patients PN is mostly caused by the administration of chemotherapeutic agents, such as oxaliplatin [4, 5]. Whereas mostly sensory PN has been associated with the type and duration of chemotherapeutic drug used, the motor and autonomic subscales may possibly be more influenced by aging [6]. The severity of PN symptoms varies greatly among patients and not everyone who received chemotherapy experiences these complaints [4]. Apart from chemotherapeutic agents, studies have shown that demographic factors [7], higher body mass index (BMI), and less physical activity [8], more comorbidities and worse functional status [9], more anxiety and depressive symptoms [10, 11], and lower health-related quality of life (HRQoL) [12] were separately associated with PN symptoms in patients with various cancer types. However, to our knowledge, none of the earlier studies have investigated the association of such factors to PN altogether in large multivariable models, thereby showing the independency of the associations.

Our aim was to investigate sociodemographic, clinical, lifestyle, and psychological factors among CRC survivors in multivariable models to describe their cross-sectional associations with sensory, motor, and autonomic PN subscales and to the PN sum score. We used this holistic approach to determine the factors that were independently associated with the PN scores, both in patients that received or did not receive chemotherapy.

## Materials and Methods

### Setting and Participants

CRC survivors diagnosed between 2000 and 2009 in the southern part of the Netherlands were recruited in the Patient Reported Outcomes Following Initial Treatment and Long-Term Evaluation of Survivorship registry, as described earlier [6, 13, 14]. The data presented in this article are based on the second wave (December 2011), in which a questionnaire on PN was included. The Medical Ethics Committee approved this study, and all participants gave written informed consent.

### Peripheral Neuropathy

PN was assessed with the European Organization for Research and Treatment of Cancer (EORTC) Quality of Life Questionnaire (QLQ) Chemotherapy-Induced PN20 [15]. This questionnaire contains three subscales assessing sensory, motor, and autonomic symptoms. Respondents are asked how often they had experienced a symptom in the past week. Items are answered on a four-point Likert scale ranging from (1) *not at all* to (4) *very much*. Scores were linearly transformed to a 0–100 scale, with higher scores indicating more complaints.

In addition, we scored the EORTC QLQ-Chemotherapy-Induced PN20 as a simple additive checklist yielding a single mean sum score, as described before [16, 17]. The sum score was derived according to the standard EORTC scoring procedures for symptom scales, with a higher score indicating higher levels of symptom burden. Outcomes were included in the analyses as continuous scores, and also dichotomized based on mean scores in the general population, in order to filter out a certain level of PN that can already be present in individuals without cancer [16]: presence of PN was defined as >3.2 for the sensory subscale, >3.8 for the motor subscale, >4.4 for the autonomic subscale, and >3.6 for the total PN score.

### Correlates

Sociodemographic correlates included age, sex, educational level, current marital status, and current employment status were collected with questionnaires. PN-related comorbidities were assessed with the adjusted Self-Administered Comorbidity Questionnaire (i.e., diabetes, rheumatoid arthritis, osteoarthritis) [18]. Clinical data related to the patient's history of CRC (i.e., years since diagnosis, tumor site [colon or rectum], tumor stage, primary treatment) were collected within the Netherlands Cancer Registry.

Self-reported data on alcohol consumption (drinks/week) and smoking status (never/former/current) were collected. Physical activity was assessed with the European Prospective Investigation into Cancer Physical Activity Questionnaire [19]. Total time spent in moderate-to-vigorous intensity physical activity (hours/week) was subtracted [20, 21]. BMI was calculated from self-reported height and weight (kg/m^2^).

Anxiety and depressive symptom scores were calculated from the Hospital Anxiety and Depression Scale, which consists of seven items for anxiety and seven items for depression (range 0–21 points), with higher scores indicating more symptoms [22]. A cut-off value of 8 for each subscale was used to identify a clinical level of anxiety or depressive symptoms [23].

Type D personality was measured with the Dutch 14-item Type D Personality Scale (DS14) [24], including seven items in the subscales for negative affectivity (i.e., tendency to experience negative emotions) and seven items for social inhibition (i.e., tendency to inhibit expression of emotions in social interaction) [25]. The 14 items of this scale are answered on a 5-point response scale ranging from (0) *false* to (4) *true,* and therefore, the two subscales range from 0 to 28.

HRQoL was assessed with the EORTC QLQ-C30 (Version 3.0) [26]. HRQoL domains were global quality of life and cognitive, emotional, physical, role, and social functioning. Symptom scales included fatigue, pain, and nausea/vomiting, and single items were constipation, diarrhea, dyspnea, insomnia, appetite loss, and financial problems. According to the EORTC guidelines, a sum score was calculated for every domain and symptom scale (0–100 points). A higher score on the domains means better functioning, whereas a higher score on the symptom scales and single items means more complaints [26].

### Statistical Analyses

All variables were described as percentages or means and standard deviations. For non-normally distributed factors the median and range were calculated, or the interquartile range for continuous variables with a fixed range. Sample characteristics of survivors with or without PN were compared using χ^2^ tests for categorical variables, independent sample *t* tests for normally distributed continuous variables, and Mann-Whitney U tests for non-normally distributed continuous variables.

Prior to data analyses, incomplete correlates were imputed with 15 multiple imputations using the Markov chain Monte Carlo method. Missing values on correlates were imputed by using information from variables available for all survivors, including the outcomes. Results from the analyses of the 15 separate datasets were pooled, correcting the standard errors of the regression coefficients for within-imputation and between-imputation variability.

Linear regression models were used to estimate the association among sociodemographic, lifestyle, clinical, and psychological correlates and the four outcomes: (1) Sensory PN, (2) Motor PN, (3) Autonomic PN, and (4) Total PN score. For each outcome, all factors were included in a multivariable model, and a parsimonious model was created with backward selection. Factors with the highest *p* values were removed one-by-one until the remaining variables all had a *p* value <0.05. With the correlates from the parsimonious models, multivariable logistic regressions were performed with the four dichotomized PN scores as the outcomes. For a few correlates, quartiles were created in order to visualize their relationship with the PN scores.

Lastly, sensitivity and subgroup analyses were performed to check the robustness of the findings. First, the backward selection procedures were performed in the subset of survivors that received chemotherapy treatment. Second, the same procedure was done while excluding survivors who had comorbid diabetes mellitus, rheumatoid arthritis, or osteoarthritis, in order to determine whether PN that may have partly been present due to these diseases differs from PN that is present in CRC survivors. Third, the analyses were repeated in the non-imputed dataset, in order to investigate whether multiple imputations could have altered the findings. All analyses were conducted using SPSS statistical software (IBM SPSS Statistics for Windows, Version 24.0; IBM Corps, Armonk, NY, USA).

## Results

Of the 1,643 respondents at the second wave, survivors with complete data on all PN subscales were included (*N* = 1,516). Of these, 65% (*N* = 980) reported PN symptoms, based on the dichotomized total PN score (Table 1). Of the total group of CRC survivors, 226 survivors had missing data on correlates.

**Fig. 1:**
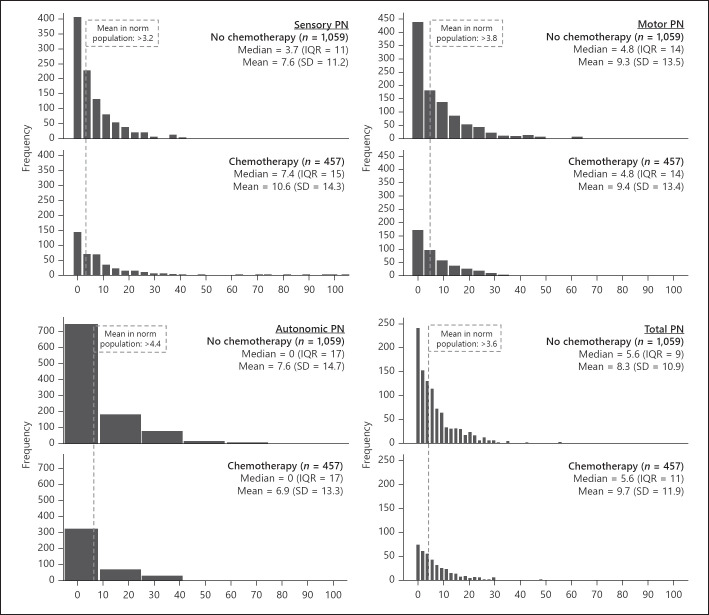
Distributions, medians (IQR), and means (SD) are shown of sensory (left upper), motor (right upper), autonomic (left lower), and total PN (right lower), both for the persons that did or did not receive chemotherapy. The dotted lines show the mean PN in the norm population, on which the dichotomizations are based for having PN. IQR, interquartile ranges; SD, standard deviation.

The median was 3.7 for sensory PN (range: 0–93), 4.8 for motor PN (range: 0–100), 0 for autonomic PN (range: 0–100), and 5.6 for the total PN score (range 0–89). The PN subscales were strongly intercorrelated (ρ = 0.37–0.66). After the PN scores were dichotomized, 959 CRC survivors (63%) had sensory PN scores > 3.2, 901 survivors (59%) had motor PN scores > 3.8, 430 survivors (28%) had autonomic PN scores > 4.4, and 980 (65%) had a total PN sum score above 3.6. The distributions of the PN scores are shown in Figure 1, including the summary statistics for both survivors that did not receive chemotherapy as the survivors that did.

The 40 correlates were entered into univariate (online suppl. Table 1; for all online suppl. material, see www.karger.com/doi/10.1159/000524037), and multivariable models (Table 2) for PN as an outcome. In the multivariable models, higher sensory PN was associated with being male, shorter time after diagnosis, receiving chemotherapy but no radiotherapy, having comorbid rheumatoid arthritis, worse cognitive, physical, and role functioning but better social functioning, more nausea/vomiting, pain, insomnia, constipation, and financial problems. These factors together explained 25% of the variance (adjusted *R*^2^) in sensory PN severity. Higher motor PN was associated with older age, female gender, no radiotherapy, having comorbid rheumatoid arthritis and osteoarthritis, more anxiety and depressive symptoms, lower cognitive, physical, and role functioning, and more nausea/vomiting, pain, and financial problems (adjusted *R*^2^ = 46%). Higher autonomic PN was higher with older age, worse cognitive and emotional functioning, fatigue, pain, and constipation (adjusted *R*^2^ = 29%). Lastly, a higher total PN sum score was associated with older age, fewer years since diagnosis, receiving chemo- but no radiotherapy, comorbid rheumatoid arthritis, higher anxiety symptoms, higher social functioning, worse cognitive, physical, and role functioning, and more fatigue, nausea/vomiting, pain, constipation, and financial problems (adjusted *R*^2^ = 43%).

**Fig. 2:**
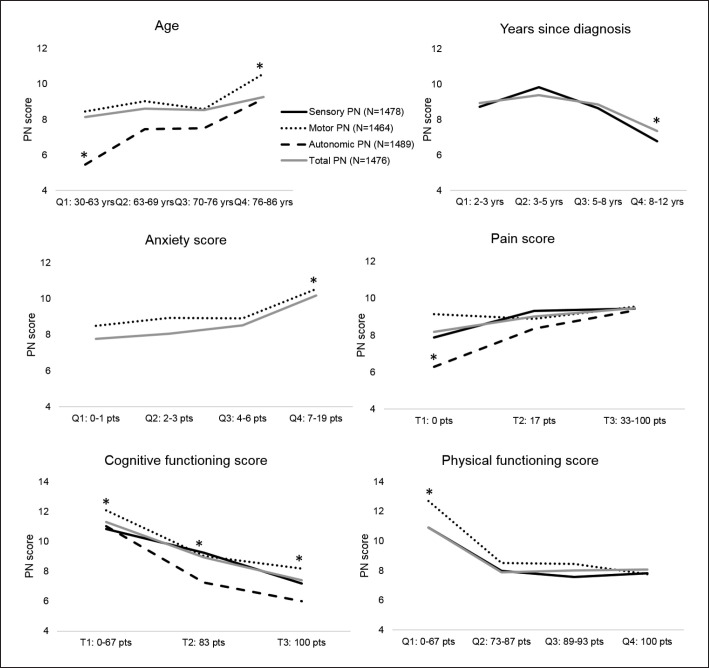
Some correlates are shown as quartiles (or tertiles for pain and cognitive functioning) on the *x*-axis versus sensory, motor, autonomic and total PN on the *y*-axis, and corrected for all other correlates in the models (as shown in Tables 2, 3). Significant differences are marked with an asterisk. In the age quartiles, Q4 was significantly higher than all other quartiles in motor and autonomic PN, but none were significant for total PN. For time since diagnosis, Q4 was significantly lower versus all quartiles in sensory and total PN. For anxiety, Q4 was significantly higher than the other quartiles in sensory and total PN. For pain, T1 was significantly lower than other tertiles in autonomic PN and significantly lower than T3 in total PN, but none were significant for sensory and motor PN. Cognitive functioning T3 was significantly lower than all other tertiles in sensory, motor, and autonomic PN, and all tertiles were different for total PN score. For physical functioning, Q1 was significantly higher than all other quartiles in sensory, motor, and autonomic PN.

Next, the analyses were repeated with the dichotomized PN outcomes (Table 3). From each domain, we selected few correlates that were associated with multiple PN outcomes and visualized them in Figure 2 as quartiles or tertiles (*x*-axis) versus the continuous PN scores (*y*-axis) with which they were associated. PN was higher in the highest age quartile (>76 years), in the first years after diagnosis, and clear dose-response relationships were visible for anxiety, pain, cognitive, and physical functioning.

Subgroup analyses in the sample that received chemotherapy (*N* = 457) showed that results were mostly consistent, but due to the smaller sample sizes there were slightly less variables in the models (data not shown). In the models of motor PN, we saw lifestyle factors that were associated with higher motor PN: smoking, physical activity, and higher BMI. This was also the case for autonomic PN and total PN scores: smoking was still significantly associated with higher scores. The associations found for the rest of the PN subscales were relatively consistent. The findings were also consistent in the group that excluded the three PN-related comorbidities (*N* = 975) or when the non-imputed datasets were used (data not shown).

## Discussion

To our knowledge, this is the first large holistic study that entered a wide range of correlates into multivariable models to determine their independent relations with sensory, motor, autonomic, and total PN in CRC survivors 2–12 years post-diagnosis. Whereas the current study aimed to assess cross-sectional correlates of PN, previous studies have mostly focused on risk factors for the development or chronicity of PN, such as oxaliplatin and other chemotherapeutic drugs, cumulative dose, genetic factors, preexisting neuropathy, and age [7]. In our study, receiving chemotherapy was related to PN as expected, and radiotherapy showed a negative association, although this finding should be interpreted with caution, as it most likely reflects differences between colon and rectum cancer patients, their treatment regimen, and disease stage. Other studies reported that age ≥60 years and initial PN can influence the duration of PN [27], and that female sex, functioning level, BMI, and baseline opioid use were associated with higher severity of oxaliplatin-induced PN [28]. Other studies have described the separate relations between PN and HRQoL, depression, perceived stress, and sleep disturbances in CRC survivors [6, 7, 12, 29, 30]. Even though these studies looked at various aspects, most studies only did univariate analyses, and thereby, do not take into account the combination of correlates. Moreover, the studies that did perform multivariable analyses, included sociodemographic, lifestyle, and psychological factors as confounding variables and only reported the effects of clinical characteristics while not reporting the effects of the other factors.

Within our study, lifestyle factors were related to PN only in the univariate analyses, such as alcohol, physical activity, and BMI, but none of them remained in the multivariable models after backward elimination. This was in contrast with another study that found in multivariable analyses that women with breast cancer with higher BMI or lower physical activity levels developed more severe PN [8]. The latter study looked specifically after taxane treatment, whereas we did not distinguish between therapies. When we selected only the survivors who received chemotherapy, we found that smoking, physical activity, and BMI were related to motor PN, and smoking was related to autonomic and total PN scores too. One explanation for this difference between the main analyses and the one with only survivors after chemotherapy is that survivors could potentially have a different lifestyle after treatment. However, survivors in our study who received chemotherapy did not differ in lifestyle patterns from the survivors without chemotherapy, except that their BMI was slightly higher. Higher motor PN levels may be related to increases in body fat and less physical fitness. Another potential explanation for the observation that these lifestyle factors did not remain in our multivariable models is the presence of psychological and HRQoL correlates that are related to both PN and to the lifestyle factors themselves.

Overall, in our multivariable models, many of the included correlates were in the psychological and HRQoL domains. Other studies have more often focused on clinical characteristics as causal factors, or psychological and HRQoL items that are impacted by or a consequence of PN. In the same dataset, we have recently published that depression and anxiety were related to PN and that this association was mediating the link between PN and fatigue [11]. These associations may be bidirectional. Psychological factors such as anxiety have also been reported to be present before PN symptoms arise [10]. One explanation is that persons with anxiety symptoms report more PN due to increased alertness to bodily symptoms and pain catastrophizing [31]. Another explanation may be that increased pro-inflammatory cytokine production is present in the anxious state, and interferes with the regeneration of nerve damage in PN [10].

Some limitations of the current study are its cross-sectional nature. Also, we did not have any information on preexisting PN, nor the type and dose of received chemotherapy, as oxaliplatin has a large effect on PN. Nevertheless, in analyses with only chemotherapy-treated survivors, we had largely consistent results. Therefore, we want to emphasize that the goal of the current paper was not to focus on risk factors or predictors of PN, but on the cross-sectional correlates. Another limitation may have been the multiple testing with 40 potential correlates in the models, and the resulting collinearity that can occur. It is important to take into account that various psychological and HRQoL factors were strongly correlated (up to ρ = 0.7), but the domains and symptoms still measure separate facets of a large complex construct of the survivor's well-being. We show both univariate analyses (online suppl. Table S1) and the multivariable models, and with the backward elimination procedure, we aimed to keep the most significantly related correlates in the models.

The strengths of this study are that it is a population-based cohort study with large sample size and high response rates, thereby making it a representative study population with a wide variety of available correlates. Moreover, the multiple imputation methods that we applied increased the statistical power. However, there is a risk of bias in longitudinal studies, especially when missing values are not random. Although the latter assumption is untestable, multiple imputations are currently the recommended strategy for imputing missing data with the least risk of bias [32]. Also, the measurement of PN symptoms was done by a well-validated questionnaire [15] that performs just as good as physician-reported PN-measures [33] and is recommended in the American Society of Clinical Oncology Clinical Practice Guidelines [34]. Furthermore, this Patient Reported Outcomes Following Initial Treatment and Long-Term Evaluation of Survivorship cohort included both short- and long-term CRC survivors ranging from 2 to 12 years post-diagnosis, thereby shedding more light on the chronicity of PN. The fact that 65% of the current sample showed PN (based on the sum score) even after a median of 5-year post-diagnosis emphasizes the importance of PN in CRC survivors.

Significant factors found in our study, even though it was cross-sectional, maybe giving clues of which persons develop PN, and in whom it gets more severe or chronic. Future research should focus on measuring PN longitudinally, starting preferably before treatments in order to disentangle the effects of cancer treatment and preexisting conditions. A longitudinal design with repeated measurements can also enable analyses of changes over time in both PN (i.e., improving over time or chronic) and its correlates.

## Conclusions

Apart from receiving chemotherapy, older age, sex, shorter time since diagnosis, PN-related comorbidities, more anxiety, and depressive symptoms, and worse HRQoL scores remained in the models after backward selection. With this multivariable approach, we found that several sociodemographic, clinical, and psychological factors were independently associated with the severity of PN. Overall, even though we included many correlates, our models explained 25%–46% of the variances in PN. These findings firstly emphasize that there are more unmeasured factors that explain whether a person develops PN. Second, future studies can take these factors into account when investigating survivors that experience PN, or in whom the various subtypes of neuropathy occur. Overall, PN can be affected by aging, sex, and stress-related factors in CRC survivors.

## Statement of Ethics

This study protocol was reviewed and approved by the Medical Ethics Committee of the Maxima Medical Centre in the Netherlands, approval number 0822. All participants gave written informed consent.

## Conflict of Interest Statement

The authors have no conflicts of interest to declare.

## Funding Sources

This work was supported by an NWO Aspasia Grant (NWO#015.012.023). Martijn J.L. Bours is supported by the Kankeronderzoekfonds Limburg as part of Health Foundation Limburg (grant No. 00005739). The funders did not have any influence on data analyses or the writing of the paper.

## Author Contributions

Data collection (Floortje Mols, Lonneke van de Poll), data analysis (Dóra Révész), interpretation of data (Dóra Révész, Cynthia Bonhof, Martijn Bours, Matty Weijenberg, and Floortje Mols), draft (Dóra Révész, Floortje Mols), revising (all), and final approval (all).

## Data Availability Statement

All data are freely available from the PROFILES registry (www.profilesregistry.nl).

## Supplementary Material

Supplementary data

## Figures and Tables

**Table 1:** Sample characteristics of survivors with or without PN, based on the total PN sum score (>3.6 for the total PN score) in non-imputed dataset

	*N*	All survivors (*N* = 1,516)	No PN (*N* = 536)	PN (*N* = 980)	*p* values
Outcomes, median (IQR)					
Sensory PN score	1,516	3.7 (11)	0 (0)	7.4 (14)	<0.001
Motor PN score	1,516	4.8 (14)	0 (0)	9.5 (14)	<0.001
Autonomic PN score	1,516	0 (17)	0 (0)	0 (17)	<0.001
Total PN sum score	1,516	5.6 (9)	0 (2)	9.3 (11)	<0.001
Sociodemographic factors, *N* (%)					
Age, years, mean (SD)	1,516	69.1 (9.4)	67.2 (9.2)	70.1 (9.4)	<0.001
Female sex	1,516	634 (41.8)	189 (35.3)	445 (45.4)	<0.001
Educational level					
High	1,504	359 (23.7)	144 (27.0)	215 (22.1)	0.03
Middle	944 (62.3)	331 (62.1)	613 (63.1)		
Low	201 (13.3)	58 (10.9)	143 (14.7)		
Employed	1,487	283 (18.7)	129 (24.4)	154 (16.1)	<0.001
Partner	1,505	1,186 (78.2)	422 (79.6)	764 (78.4)	0.57
Clinical characteristics, *N* (%)					
Years since diagnosis, years, md (range)	1,516	5.2 (2–12)	6.0 (2–12)	4.8 (2–12)	<0.001
Tumor location (colon vs. rectum)	1,516	896 (59.1)	307 (57.3)	589 (60.1)	0.29
TNM stage					
I	1,478	453 (29.9)	183 (35.1)	270 (28.2)	0.009
II	540 (35.6)	189 (36.3)	351 (36.7)		
III	435 (28.7)	138 (26.5)	297 (31.0)		
IV	50 (3.3)	11 (2.1)	39 (4.1)		
Chemotherapy	1,516	457 (30.1)	141 (26.3)	316 (32.2)	0.02
Radiotherapy	1,516	493 (32.5)	183 (34.1)	310 (31.6)	0.32
Diabetes mellitus	1,516	202 (13.3)	63 (11.8)	139 (14.2)	0.18
Osteoarthritis	1,516	365 (24.1)	68 (12.7)	297 (30.3)	<0.001
Rheumatoid arthritis	1,516	76 (5.0)	10 (1.9)	66 (6.7)	<0.001
Lifestyle factors					
Alcohol, drinks/week, md (range)	1,501	4.0 (0–100)	5.0 (0–50)	4.0 (0–100)	0.006
Smoking, *N* (%)					
Nonsmoker	1,508	468 (30.9)	172 (32.1)	296 (30.5)	0.80
Former smoker	882 (58.2)	308 (57.5)	574 (59.1)		
Current smoker	158 (10.4)	56 (10.4)	102 (10.5)		
Physical activity, h/wk, md (range)	1,438	10.0 (0–50)	11.0 (0–47)	9.5 (0–50)	<0.001
BMI, kg/m^2^, mean (SD)	1,508	26.8 (4.5)	26.5 (4.2)	27.0 (4.6)	0.03
Psychological factors, median (IQR)					
Anxiety, *N* (%)	1,512	293 (19.3)	40 (7.5)	253 (25.9)	<0.001
Depression, *N* (%)	1,513	262 (17.3)	36 (6.7)	226 (23.1)	<0.001
Negative affectivity score	1,497	6 (9)	3 (9)	7 (10)	<0.001
Social inhibition score	1,503	7 (10)	6 (9)	8 (10)	<0.001
HRQoL domains and single-item scores					
Global quality of life	1,512	83 (25)	83 (17)	75 (17)	<0.001
Cognitive functioning	1,512	100 (17)	100 (17)	83 (33)	<0.001
Emotional functioning	1,509	100 (17)	100 (8)	92 (25)	<0.001
Physical functioning	1,512	87 (27)	93 (13)	83 (27)	<0.001
Role functioning	1,508	100 (33)	100 (0)	83 (33)	<0.001
Social functioning	1,510	100 (17)	100 (0)	100 (33)	<0.001
Fatigue	1,508	11 (33)	0 (22)	22 (22)	<0.001
Nausea/vomiting	1,512	0 (0)	0 (0)	0 (0)	<0.001
Pain	1,513	0 (33)	0 (0)	17 (33)	<0.001
Dyspnea	1,500	0 (33)	0 (0)	0 (33)	<0.001
Insomnia	1,507	0 (33)	0 (33)	0 (33)	<0.001
Appetite loss	1,508	0 (0)	0 (0)	0 (0)	<0.001
Constipation	1,499	0 (0)	0 (0)	0 (0)	<0.001
Diarrhea	1,498	0 (33)	0 (0)	0 (33)	<0.001
Financial problems	1,510	0 (0)	0 (0)	0 (0)	<0.001

Means and SDs, and numbers and percentages, are shown for normally distributed variables, and medians and ranges or interquartile ranges for non-normally distributed variables. Ranges are given for variables that do not have a fixed range (such as questionnaires). IQR, interquartile range; SD, standard deviation.

**Table 2:** Multivariate analyses with backward selected factors (until *p* values <0.05) for each PN subscale and total PN score in imputed dataset (*N* = 1,516)

	Sensory PN score	Motor PN score		Autonomic PN score		Total PN sum score	
	β (SE)	*p* value	β (SE)	*p* value	β (SE)	*p* value	β (SE)	*p* value
Sociodemographic factors								
Age (years)			0.11 (0.03)	<0.001	0.15 (0.03)	<0.001	0.08 (0.03)	0.002
Sex (ref = male)	−2.00 (0.57)	0.001	2.22 (0.54)	<0.001				
Educational level (ref = high)								
Middle								
Low								
Employment (ref = employed) Partner (ref = partner)								
Clinical factors								
Years since diagnosis (years)	−0.33 (0.10)	0.001					−0.22 (0.08)	0.005
Tumor location (ref = colon vs. rectum cancer)								
Tumor stage (ref = stage 1)								
II								
III								
IV								
Chemotherapy (ref = no)	2.69 (0.60)	<0.001					1.41 (0.49)	0.004
Radiotherapy (ref = no)	−1.28 (0.59)	0.03	−1.39 (0.55)	0.01			−1.16 (0.47)	
Diabetes mellitus (ref = no)								
Rheumatoid arthritis (ref = no)	4.40 (1.29)	0.001	6.38 (1.21)	<0.001			5.03 (1.03)	<0.001
Osteoarthritis (ref = no)			1.29 (0.65)	0.048				
Lifestyle factors								
Alcohol (drinks/week)								
Smoking (ref = never smoker)								
Former smoker								
Current smoker								
Physical activity (hours/week)^a^								
BMI (kg/m^2^)								
Psychological and HRQoL factors								
Anxiety (ref = no)^b^		1.90 (0.75)		0.01			1.81 (0.61)	0.003
Depression (ref = no)^b^		1.97 (0.80)		0.01				
Negative affectivity score								
Social inhibition score								
Global quality of life^c^								
Cognitive functioning	−0.10 (0.02)	<0.001	−0.12 (0.02)	<0.001	−0.18 (0.02)	<0.001	−0.11 (0.01)	<0.001
Emotional functioning					−0.06 (0.02)	0.01		
Physical functioning	−0.09 (0.02)	<0.001	−0.17 (0.02)	<0.001			−0.10 (0.02)	<0.001
Role functioning	−0.05 (0.02)	0.002	−0.08 (0.02)	<0.001			−0.06 (0.02)	<0.001
Social functioning	0.06 (0.02)	0.003					0.04 (0.02)	0.02
Fatigue					0.11 (0.02)	<0.001	0.04 (0.02)	0.02
Nausea/vomiting	0.08 (0.03)	0.005	0.07 (0.03)	0.01			0.07 (0.02)	0.004
Pain	0.06 (0.02)	<0.001	0.04 (0.02)	0.01	0.08 (0.02)	<0.001	0.05 (0.01)	<0.001
Dyspnea								
Insomnia Appetite loss	0.02 (0.01)	0.03									
Constipation	0.03 (0.02)	0.03				0.09 (0.02)		<0.001	0.04 (0.01)		0.004
Diarrhea											
Financial problems	0.06 (0.02)	<0.001	0.08 (0.02)		<0.001				0.07 (0.01)		<0.001
Explained variance (adjusted *R*^2^) (%)		25		46			29			43	

a Physical activity was defined as the hours of moderate-to-vigorous intensity physical activity per week.

b Anxiety and depression were defined as scoring ≥8 on the HADS score.

c HRQoL, health-related quality of life, included domains, symptom scales, and single items.

**Table 3:** Multivariable logistic regression with selected factors for each dichotomized PN subscale and total PN score in an imputed dataset (*N* = 1,516)

	Sensory PN (>3.2)^a^ OR [95% CI]	*p* value	Motor PN (>3.8)^a^ OR [95% CI]	*p* value	Autonomic PN (>4.4)^a^ OR [95% CI]	*p* value	Total PN (>3.6)^a^ OR [95% CI]	*p* value
Sociodemographic factors								
Age (years)			1.22 [1.07–1.40]	0.003	1.38 [1.21–1.57]	<0.001	1.35 [1.18–1.55]	<0.001
Sex (ref = male)	0.80 [0.64–1.02]	0.07	1.76 [1.37–2.26]	<0.001				
Clinical factors								
Years since diagnosis (years)	0.96 [0.93–1.00]	0.06					0.94 [0.90–0.98]	0.004
Chemotherapy (ref = no)	1.31 [1.02–1.67]	0.04					1.52 [1.16–2.00]	0.002
Radiotherapy (ref = no)	0.77 [0.61–0.98]	0.03	0.85 [0.66–1.10]	0.22			0.90 [0.69–1.17]	0.43
Rheumatoid arthritis (ref = no)	1.38 [0.75–2.53]	0.30	3.08 [1.33–7.17]	0.01			2.17 [1.04–4.55]	0.04
Osteoarthritis (ref = no)			2.10 [1.52–2.92]	<0.001				
Psychological and HRQoL factors								
Anxiety (ref = no)^b^			1.46 [1.00–2.12]	0.049			2.15 [1.44–3.21]	<0.001
Depression (ref = no)^b^			1.27 [0.84–1.92]	0.26				
Cognitive functioning (+18.9)^c^	0.62 [0.52–0.73]	<0.001	0.66 [0.55–0.78]	<0.001	0.72 [0.62–0.83]	<0.001	0.60 [0.49–0.72]	<0.001
Emotional functioning (+17.6)^c^					0.93 [0.79–1.08]	0.33		
Physical functioning (+19.1)^c^ Role functioning (+24.9)^c^	0.76 [0.63–0.92]	0.01	0.55 [0.43–0.68]	<0.001			0.58 [0.45–0.74]	<0.001
	0.86 [0.70–1.06]	0.16	0.71 [0.57–0.90]	0.004			0.82 [0.64–1.06]	0.13
Social functioning (+20.5)^c^	1.17 [0.98–1.40]	0.09					1.15 [0.92–1.42]	0.22
Fatigue (+22.3)^d^					1.60 [1.35–1.89]	<0.001	1.17 [0.93–1.48]	0.18
Nausea/vomiting (+10.8)^d^	1.08 [0.91–1.28]	0.36	1.06 [0.88–1.29]	0.53			1.16 [0.93–1.46]	0.19
Pain (+24.0)^d^	1.27 [1.06–1.53]	0.01	1.11 [0.90–1.37]	0.35	1.26 [1.09–1.46]	0.002	1.37 [1.10–1.72]	0.01
Insomnia (+26.4)^d^	1.17 [1.01–1.35]	0.03						
Constipation (18.8)^d^	1.10 [0.96–1.26]	0.17			1.25 [1.11–1.42]	<0.001	1.13 [0.97–1.32]	0.11
Financial problems (+17.7)^d^	1.07 [0.92–1.25]	0.36	1.11 [0.95–1.30]	0.21			1.11 [0.93–1.32]	0.26

a PN scores were dichotomized based on mean scores in the general population: >3.2 for sensory subscale, >3.8 for motor subscale, >4.4 for autonomic subscale, and >3.6 for total PN score (13).

b Anxiety and depression were defined as scoring ≥8 on the HADS score.

c HRQoL domains are entered as standardized scores with SDs shown between brackets: higher scores represent better functioning.

d HRQoL symptom scales and single items are entered as standardized scores with SDs shown between brackets: higher scores represent more complaints.
