# Supplementary material for: Sociodemographic, Clinical, Lifestyle, and Psychological Correlates of Peripheral Neuropathy among 2- to 12-Year Colorectal Cancer Survivors
Source: Oncol Res Treat. 2022 Mar 18;45(9):480–93. doi: 10.1159/000524037 (PMC9533432; doi:10.1159/000524037)
Supplement: Supplementary file 1 — Supplementary data [file ort-0045-0480-s01.docx]

| **Supplemental Table 1: Univariate analyses for each peripheral neuropathy (PN) subscale and total PN score in imputed dataset (N=1516)** | | | | | | | | | |
| --- | --- | --- | --- | --- | --- | --- | --- | --- | --- |
|  | | **Sensory PN score** | | **Motor PN score** | | **Autonomic PN score** | | **Total PN sum score** | |
|  |  | **β (SE)** | **p** | **β (SE)** | **p** | **β (SE)** | **p** | **β (SE)** | **p** |
| **Sociodemographic factors** | | | | | | | | | |
| Age (years) | | 0.04 (0.03) | .27 | 0.19 (0.04) | **<.001** | 0.14 (0.04) | **<.001** | 0.11 (0.03) | **<.001** |
| Sex (ref=male) | | -0.48 (0.64) | .46 | 4.28 (0.69) | **<.001** | 1.63 (0.74) | **.03** | 1.61 (0.58) | **.006** |
| Educational level (ref=high) | Middle | 0.12 (0.76) | .87 | 1.82 (0.85) | **.03** | -0.06 (0.89) | .95 | 0.76 (0.70) | .28 |
|  | Low | 1.46 (1.08) | .18 | 4.23 (1.23) | **.001** | 2.07 (1.27) | .10 | 2.61 (1.00) | **.01** |
| Employment (ref=employed) | | 1.18 (0.81) | .15 | 4.69 (0.88) | **<.001** | 2.83 (0.94) | **.002** | 2.73 (0.73) | **<.001** |
| Partner (ref=partner) | | 0.26 (0.78) | .74 | 2.49 (0.85) | **.003** | 1.82 (0.90) | **.04** | 1.30 (0.71) | .07 |
| **Clinical factors** | | | | | | | | | |
| Years since diagnosis (years) | | -0.49 (0.11) | **<.001** | -0.35 (0.12) | **.005** | -0.09 (0.13) | .51 | -0.39 (0.10) | **<.001** |
| Tumor location (ref=colon vs. rectum cancer) | | -0.72 (0.64) | .26 | -1.38 (0.70) | **.05** | -0.62 (0.75) | .41 | -1.00 (0.58) | .10 |
| Tumor stage (ref=stage I) | II | 0.46 (0.76) | .54 | 1.57 (0.84) | .06 | 1.26 (0.89) | .16 | 0.98 (0.70) | .16 |
|  | III | 2.33 (0.80) | **.004** | 0.59 (0.89) | .51 | -0.25 (0.94) | .79 | 1.37 (0.74) | .06 |
|  | IV | 2.11 (1.77) | .24 | 1.36 (1.82) | .46 | 1.16 (1.94) | .55 | 1.71 (1.57) | .28 |
| Chemotherapy (ref=no) | | 3.05 (0.68) | **<.001** | 0.02 (0.75) | .98 | -0.74 (0.80) | .36 | 1.45 (0.63) | **.02** |
| Radiotherapy (ref=no) | | -1.19 (0.67) | .08 | -1.23 (0.74) | .10 | -0.76 (0.78) | .33 | -1.16 (0.61) | .06 |
| Diabetes mellitus (ref=no) | | 2.63 (0.93) | **.004** | 1.82 (1.02) | .07 | 2.21 (1.08) | **.04** | 2.27 (0.84) | **.007** |
| Osteoarthritis (ref=no) | | 4.28 (0.73) | **<.001** | 7.35 (0.79) | **<.001** | 4.49 (0.85) | **<.001** | 5.50 (0.66) | **<.001** |
| Rheumatoid arthritis (ref=no) | | 9.23 (1.43) | **<.001** | 13.78 (1.55) | **<.001** | 7.21 (1.67) | **<.001** | 10.78 (1.29) | **<.001** |
| **Lifestyle factors** | | | | | | | | | |
| Alcohol (drinks/week) | | -0.03 (0.04) | .44 | -0.08 (0.04) | **.05** | -0.07 (0.04) | .12 | -0.05 (0.03) | .12 |
| Smoking (ref=never smoker) | Former smoker | 0.68 (0.70) | .33 | -0.45 (0.77) | .56 | -1.30 (0.82) | .11 | 0.02 (0.64) | .97 |
|  | Current smoker | 1.35 (1.13) | .23 | 1.36 (1.23) | .27 | 1.88 (1.31) | .15 | 1.41 (1.02) | .17 |
| Physical activity (hours/week)^a^ | | -0.06 (0.04) | .11 | -0.18 (0.04) | **<.001** | -0.11 (0.04) | **.01** | -0.11 (0.03) | **.001** |
| Body mass index (kg/m^2^) | | 0.21 (0.07) | **.003** | 0.12 (0.08) | .13 | 0.12 (0.08) | .16 | 0.16 (0.06) | **.01** |
| **Psychological and health-related quality of life (HRQoL) factors** | | | | | | | | | |
| Anxiety (ref=no) ^b^ | | 6.65 (0.78) | **<.001** | 10.26 (0.83) | **<.001** | 9.18 (0.90) | **<.001** | 8.34 (0.70) | **<.001** |
| Depression (ref=no) ^b^ | | 7.80 (0.81) | **<.001** | 12.48 (0.86) | **<.001** | 9.95 (0.94) | **<.001** | 9.86 (0.72) | **<.001** |
| Negative affectivity score | | 0.38 (0.05) | **<.001** | 0.57 (0.06) | **<.001** | 0.58 (0.06) | **<.001** | 0.48 (0.05) | **<.001** |
| Social inhibition score | | 0.21 (0.05) | **<.001** | 0.31 (0.06) | **<.001** | 0.35 (0.06) | **<.001** | 0.26 (0.05) | **<.001** |
| Global quality of life ^c^ | | -0.21 (0.02) | **<.001** | -0.31 (0.02) | **<.001** | -0.27 (0.02) | **<.001** | -0.25 (0.01) | **<.001** |
| Cognitive Functioning | | -0.22 (0.02) | **<.001** | -0.32 (0.02) | **<.001** | -0.33 (0.02) | **<.001** | -0.27 (0.01) | **<.001** |
| Emotional Functioning | | -0.20 (0.02) | **<.001** | -0.30 (0.02) | **<.001** | -0.29 (0.02) | **<.001** | -0.25 (0.02) | **<.001** |
| Physical Functioning | | -0.24 (0.02) | **<.001** | -0.41 (0.02) | **<.001** | -0.27 (0.02) | **<.001** | -0.31 (0.01) | **<.001** |
| Role Functioning | | -0.19 (0.01) | **<.001** | -0.29 (0.01) | **<.001** | -0.21 (0.01) | **<.001** | -0.23 (0.01) | **<.001** |
| Social Functioning | | -0.16 (0.02) | **<.001** | -0.28 (0.02) | **<.001** | -0.25 (0.02) | **<.001** | -0.22 (0.01) | **<.001** |
| Fatigue | | 0.22 (0.01) | **<.001** | 0.32 (0.01) | **<.001** | 0.28 (0.02) | **<.001** | 0.26 (0.01) | **<.001** |
| Nausea / Vomiting | | 0.27 (0.03) | **<.001** | 0.37 (0.03) | **<.001** | 0.36 (0.03) | **<.001** | 0.32 (0.03) | **<.001** |
| Pain | | 0.19 (0.01) | **<.001** | 0.28 (0.01) | **<.001** | 0.23 (0.01) | **<.001** | 0.23 (0.01) | **<.001** |
| Dyspnea | | 0.13 (0.01) | **<.001** | 0.20 (0.01) | **<.001** | 0.16 (0.02) | **<.001** | 0.16 (0.01) | **<.001** |
| Insomnia | | 0.11 (0.01) | **<.001** | 0.16 (0.01) | **<.001** | 0.12 (0.01) | **<.001** | 0.13 (0.01) | **<.001** |
| Appetite loss | | 0.16 (0.02) | **<.001** | 0.27 (0.02) | **<.001** | 0.21 (0.02) | **<.001** | 0.21 (0.02) | **<.001** |
| Constipation | | 0.09 (0.02) | **<.001** | 0.13 (0.02) | **<.001** | 0.17 (0.02) | **<.001** | 0.11 (0.02) | **<.001** |
| Diarrhea | | 0.07 (0.02) | **<.001** | 0.12 (0.02) | **<.001** | 0.10 (0.02) | **<.001** | 0.09 (0.01) | **<.001** |
| Financial Problems | | 0.17 (0.02) | **<.001** | 0.23 (0.02) | **<.001** | 0.17 (0.02) | **<.001** | 0.19 (0.02) | **<.001** |
| **Footnotes**:  ^a^ Physical activity was defined as the hours of moderate-to-vigorous intensity physical activity per week;  ^b^ Anxiety and depression were defined as scoring ≥8 on the HADS score;  ^c^ HRQoL = Health-related quality of life, included domains, symptom scales and single items | | | | | | | | | |
